# Alpha-6 integrin promotes radioresistance of glioblastoma by modulating DNA damage response and the transcription factor Zeb1

**DOI:** 10.1038/s41419-018-0853-x

**Published:** 2018-08-29

**Authors:** Aline Kowalski-Chauvel, Anouchka Modesto, Valerie Gouaze-andersson, Laurent Baricault, Julia Gilhodes, Caroline Delmas, Anthony Lemarie, Christine Toulas, Elizabeth Cohen-Jonathan-Moyal, Catherine Seva

**Affiliations:** 1 INSERM UMR.1037-Cancer Research Center of Toulouse (CRCT)/University Paul Sabatier, Toulouse III, France; 2 IUCT-oncopole, Toulouse, France

## Abstract

Radiotherapy is the cornerstone of glioblastoma (GBM) standard treatment. However, radioresistance of cancer cells leads to an inevitable recurrence. In the present study, we showed that blocking α6-integrin in cells derived from GBM biopsy specimens cultured as neurospheres, sensitized cells to radiation. In cells downregulated for α6-integrin expression, we observed a decrease in cell survival after irradiation and an increase in radio-induced cell death. We also demonstrated that inhibition of α6-integrin expression affects DNA damage checkpoint and repair. Indeed, we observed a persistence of γ-H2AX staining after IR and the abrogation of the DNA damage-induced G2/M checkpoint, likely through the downregulation of the checkpoint kinase CHK1 and its downstream target Cdc25c. We also showed that α6-integrin contributes to GBM radioresistance by controlling the expression of the transcriptional network ZEB1/OLIG2/SOX2. Finally, the clinical data from TCGA and Rembrandt databases demonstrate that GBM patients with high levels of the five genes signature, including α6-integrin and its targets, CHK1, ZEB1, OLIG2 and SOX2, have a significantly shorter overall survival. Our study suggest that α6-integrin is an attractive therapeutic target to overcome radioresistance of GBM cancer cells.

## Introduction

Glioblastoma (GBM) is the most lethal primary brain tumor in adult. Despite treatments including surgical resection, radiotherapy and adjuvant chemotherapy, median survival remains low around 20 months^1^. These tumors are particularly chemo- and radioresistant and are characterized by a high capacity to invade surrounding normal brain^2^. Interactions between extracellular matrix (ECM) and cancer cells have been shown to play an important role in resistance to anticancer therapies and invasion. Integrins are major receptors involved in cell–matrix adhesion. They are composed of two transmembrane glycoproteins α and β, which interact with several ECM components to regulate numerous cellular effects including proliferation, survival and invasion^3,4^. Among the integrin subunits strongly expressed in GBM, α6 is of particular interest^5,6^. Although α6-integrin is weakly expressed in normal brain, its expression is high in embryonic and adult normal neural stem cells and is involved in the growth regulation of these particular cells^7,8^. In addition, α6-integrin is recognized as an enrichment marker for GBM stem cells (GSCs) and plays a crucial role in their capacity of self-renewal, proliferation and tumor formation^9^. α6-Integrin is also a crucial regulator of GBM cells migration and invasion^10^. α6-Integrin is also necessary for the high tumorigenicity of cancer stem cells from other tumors including cervical and breast tumors^11–13^. In addition, α6-integrin is overexpressed in more differentiated cancer cells from, breast, prostate or colorectal tumors and plays an important role in the survival and metastatic potential of these cells^14–16^.

Radiotherapy is the cornerstone of initial treatment but local recurrence that occurs in almost all cases highlights the high radioresistance of GBM and GSC in particular. An attractive strategy to reduce the high risk of recurrence could be the increase of radiotherapy cytotoxic effect on tumor cells by specifically targeting factors involved in radioresistance. However to date, there is no targeted therapy, available in clinic as a standard treatment, to radiosensitize GBM. Therefore, there is a need for better understanding radioresistance mechanisms and to identify new factors that might be targeted to increase the response to radiotherapy. In this study, we therefore tested whether the overexpression of α6-integrin observed in tumor cells derived from human GBM biopsy specimens has a functional role in mediating resistance to radiotherapy. We also deciphered the possible mechanisms by which this integrin regulates GBM radioresistance.

Our data demonstrate that targeting α6-integrin increases radiosensitization of GBM, they identify a novel role for α6-integrin in tumor radioresistance and suggest that this is an attractive therapeutic target to overcome radioresistance of GBM cancer cells.

## Results

### Downregulation of α6-integrin gene expression radiosensitizes tumor cells derived from human GBM biopsy specimens

**Fig. 1:**
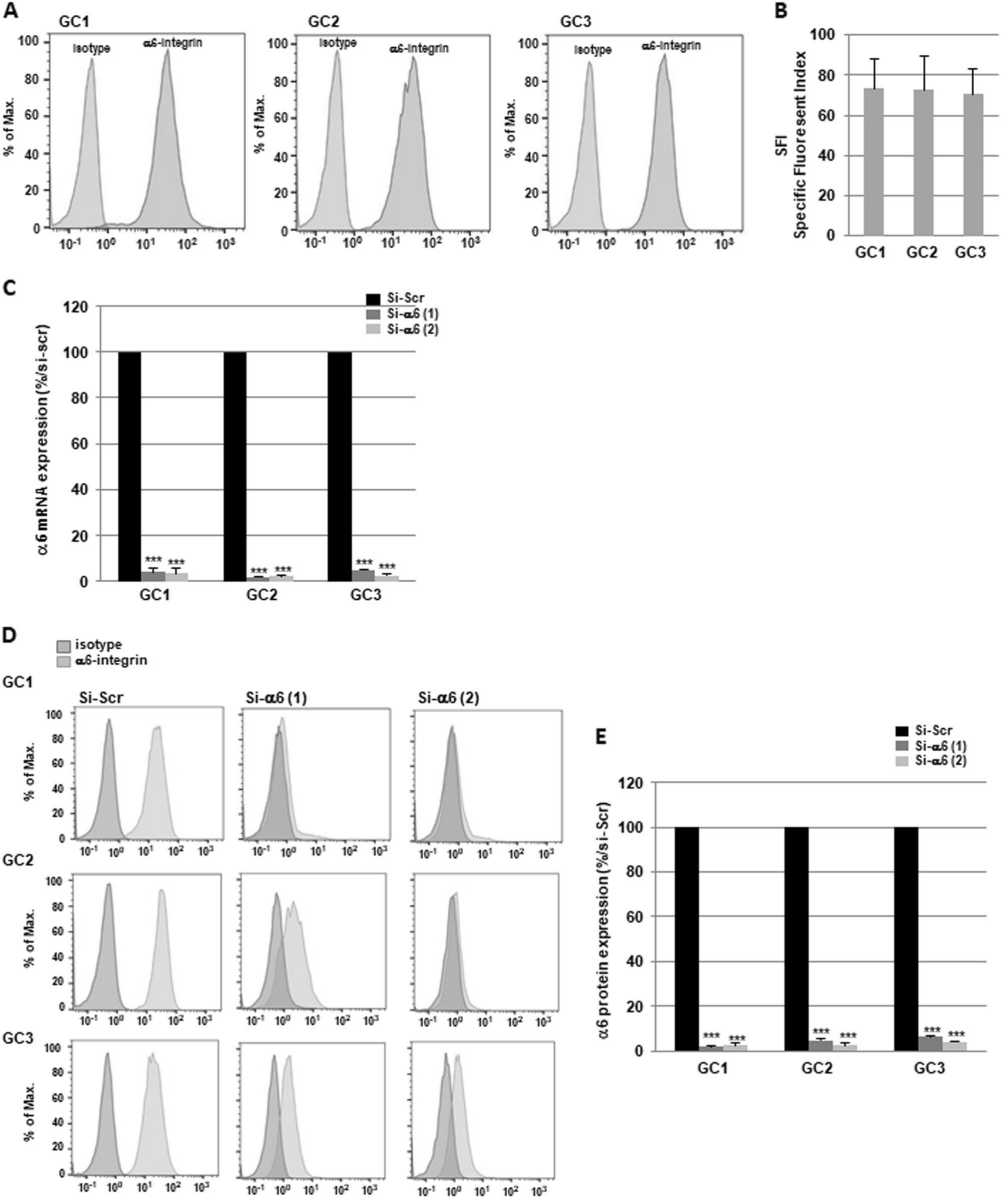
Expression of α6-integrin in tumor cells derived from human glioblastoma biopsy specimens and downregulation by specific α6-integrin siRNA. **a** α6-Integrin protein expression was analyzed by flow cytometry in tumor cells derived from three human GBM biopsy specimens (GC1, GC2 and GC3) cultured as neurospheres. Images are representative of three independent experiments. **b** The SFI allowed to evaluate α6-integrin expression level (see Materials and methods section). Results are expressed as the means ± S.E.M. of three independent experiments. **c**–**e** Cells were transfected with two different α6-integrin siRNA (si-α6 (1) or si-α6 (2)) or a scramble control (si-Scr). **c** α6-Integrin mRNA expression was analyzed by real-time PCR. **d**, **e** α6-Integrin protein expression was analyzed by flow cytometry. **e** Quantifications of three experiments are presented as means ± SD. ***p < 0.001;

To determine the role of α6-integrin subunit in GBM radioresistance, we used tumor cells derived from three human GBM biopsy specimens (GC1, GC2 and GC3) cultured as primary neurospheres that express high levels of α6-integrin subunit as shown in Fig. 1a, b. We also observed in the three primary cell lines a high expression of several stem cell markers Sox2, Olig2 and Nestin (Supplemental Fig. S1). In contrast, the differentiation markers, such as Tuj-1, were weakly expressed (Supplemental Fig. S1) or not expressed (GFAP, Glial fibrillary acidic protein, data not shown). It has been previously reported that irradiation (IR) can induce pro-survival signaling pathways and the expression of factors involved in radioresistance^17^. Therefore, before evaluating if α6-integrin subunit overexpression is functionally relevant for mediating radioresistance, we investigated whether IR could increase its expression. GBM neurospheres were exposed to different radiation doses administered as a unique dose (10 Gy) or by daily multifractions of 4 × 2.5 Gy. However, we did not observe any effect of IR on α6-integrin expression (data not shown).

To analyze the role of α6-integrin in GBM radioresistance, two different specific small interfering RNA (siRNA) were used to knockdown α6-integrin expression. Both siRNA inhibited significantly α6-integrin mRNA expression compared with a scramble control in the three neurospheres cell lines (Fig. 1c). Inhibition of α6-integrin expression by the two specific siRNA was also confirmed at the protein level by FACS (fluorescence activated cell sorting) analysis (Fig. 1d, e).

**Fig. 2:**
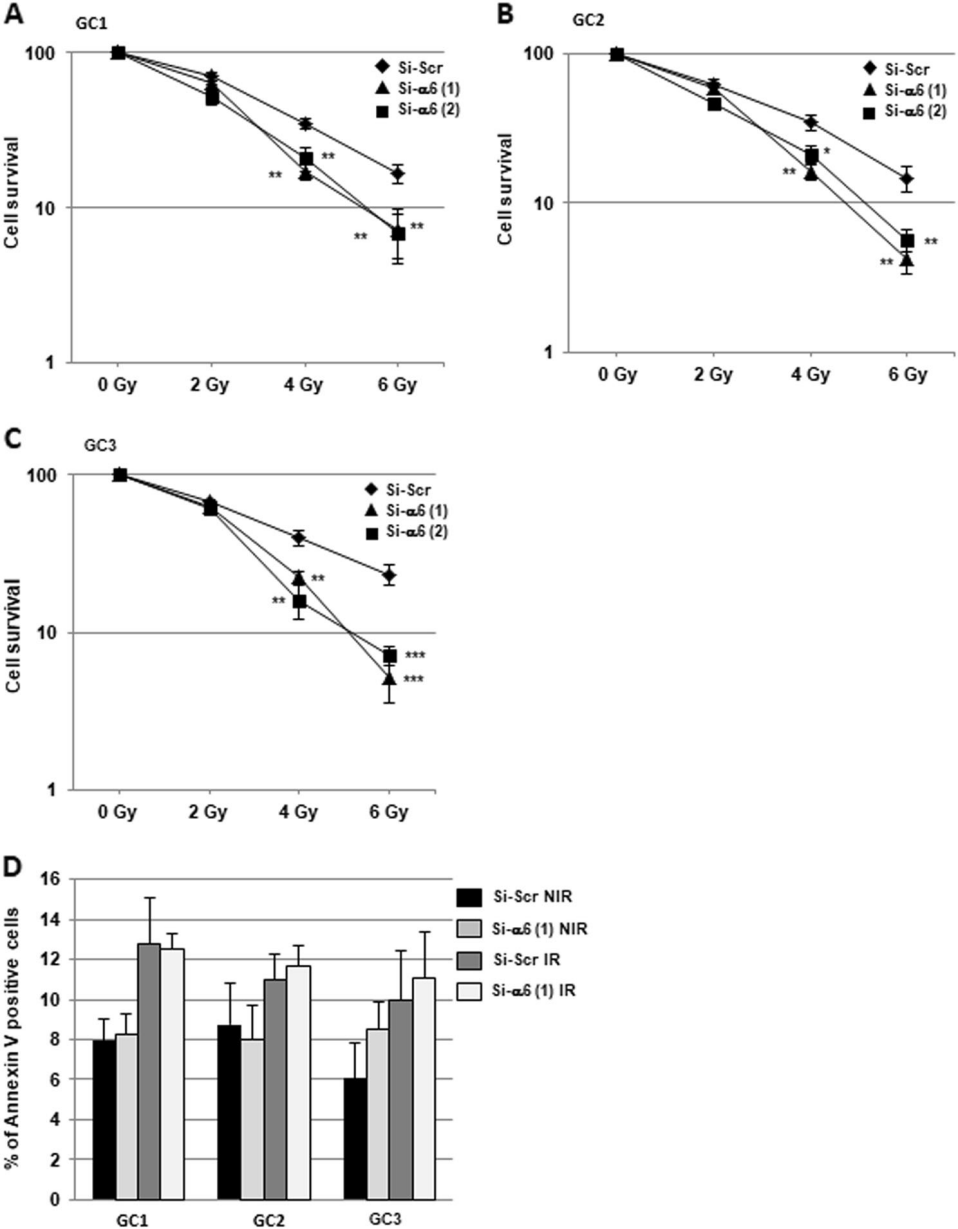
Downregulation of α6-integrin gene expression radiosensitizes tumor cells derived from human GBM biopsy specimens. Cells derived from three GBM biopsy specimens (GC1, GC2 and GC3) were transfected with two different α6-integrin siRNA (si-α6 (1) or si-α6 (2)) or a scramble control (si-Scr). **a**–**c** Cells were analyzed in 3D clonogenic assays as described in Materials and methods section. **d** Percentage of Annexin V-positive cells were analyzed by flow cytometry in cells non-irradiated (NIR) or 48 h post-irradiation (6 Gy, IR) as described in “Materials and methods section”. Quantifications of three experiments are presented as means ± SD. ***p < 0.001; **0.001 < *p* < 0.01; *0.01 < *p* < 0.05

To determine whether α6-integrin affects radiation sensitivity, we performed three-dimensional (3D) clonogenic survival assays with increasing doses of IR (Fig. 2a–c). The survival fractions after IR were significantly decreased in all GBM cell lines transfected with the specific α6-integrin siRNA compared with the control siRNA, indicating that downregulation of α6-integrin mRNA expression radiosensitizes tumor cells derived from human GBM biopsy specimens.

### Targeting α6-integrin expression modulates DNA damage response

**Fig. 3:**
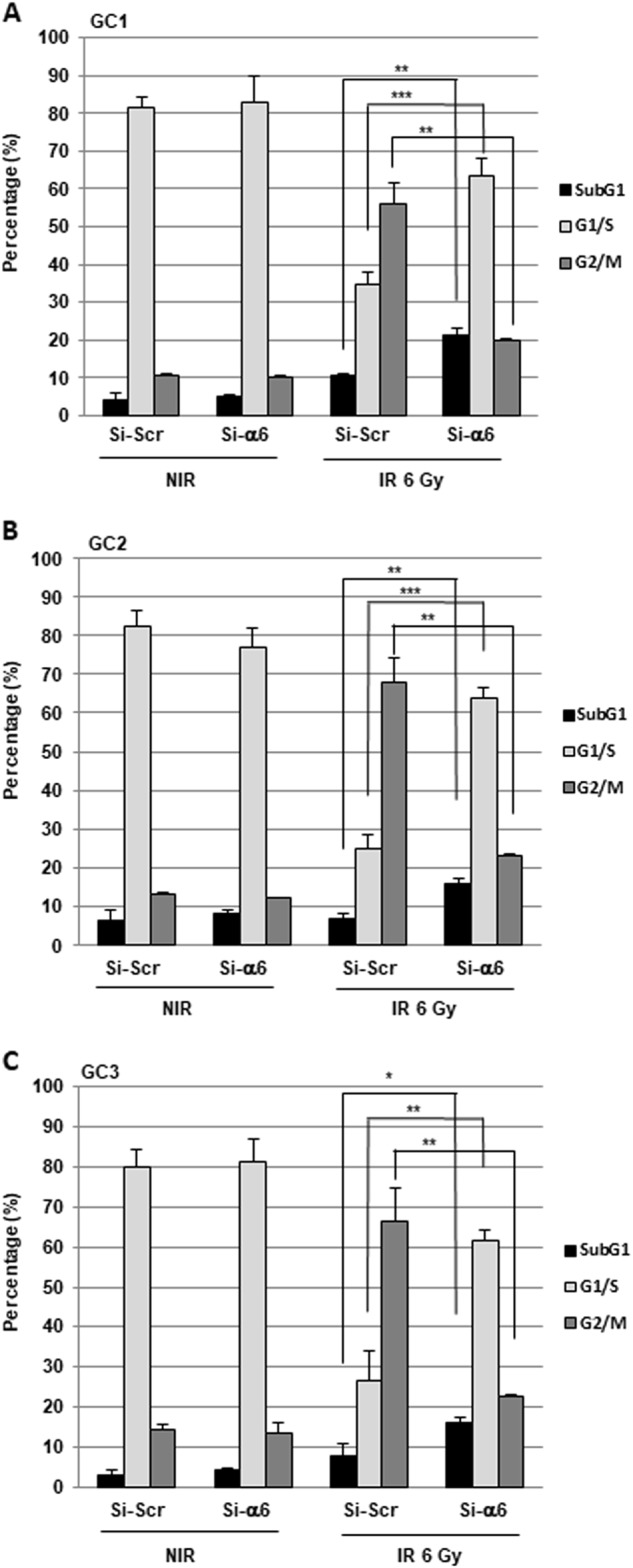
Cell cycle analysis. Cells derived from three GBM biopsy specimens (GC1, GC2 and GC3) were transfected with an α6-integrin siRNA (si-α6) or a scramble control (si-Scr). Forty-eight hours post-irradiation (6 Gy), propidium iodide staining was performed as described in “Materials and methods section” and the DNA content was analyzed by flow cytometry. Percentage of cells in sub-G1, G1/S and G2/M phases were quantified by the BD Accuri C6 software. Quantifications of three experiments are presented as means ± SD. ***p < 0.001; **0.001 < *p* < 0.01; *0.01 <* p* < 0.05.

Next, we investigated the cellular mechanisms by which α6-integrin downregulation leads to GBM cells radiosensitization. We first analyzed whether the decrease in cell survival observed when we combined α6-integrin siRNA and IR was due to an increase in apoptosis. Analysis of annexin V-positive cells following IR did not show any significant effect of IR alone or in combination with α6-integrin siRNA on apoptosis (Fig. 2d and Supplemental Fig. S2). However, we observed that α6-integrin downregulation resulted in a significant increase of radio-induced cell death, measured after 6 Gy of IR, as shown by an increase of the sub-G1 fraction in cells expressing α6-integrin siRNA compared with those expressing the scramble control (Fig. 3a–c). As expected, we also observed in the three primary cell lines, transfected with the scramble control, an arrest in the G2/M phase following IR necessary to allow IR-induced DNA repair (Fig. 3a-c). Interestingly, downregulation of α6-integrin in irradiated cells led to a high decrease of the G2/M phase that might be responsible of a less efficient repair (Fig. 3a–c) and might explain the increase in radiosensitivity when α6-integrin expression is knocked-down.

**Fig. 4:**
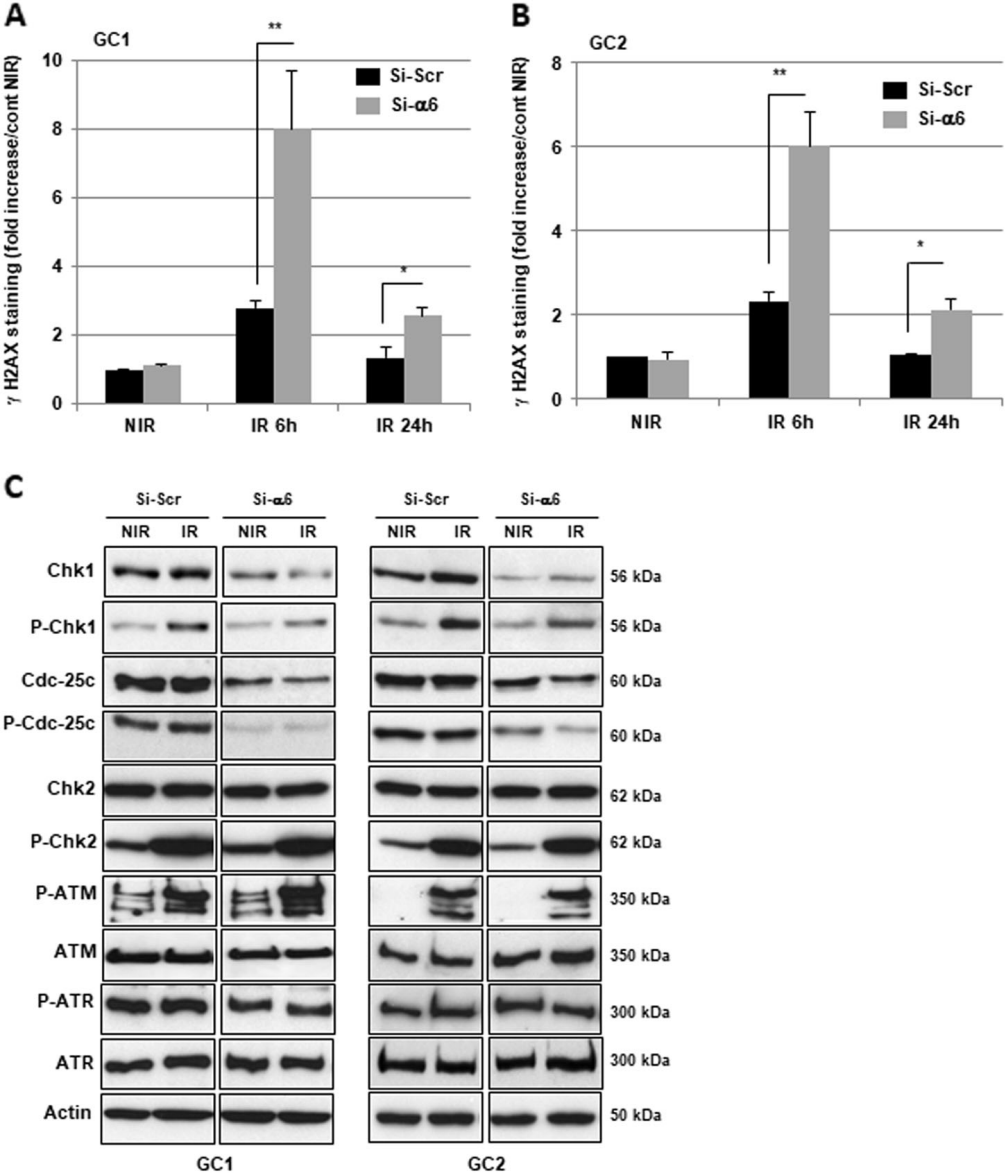
Targeting α6-integrin expression modulates DNA damage response. Cells derived from two GBM biopsy specimens (GC1 and GC2) were transfected with an α6-integrin siRNA (si-α6) or a scramble control (si-Scr). **a** Different DNA damage response proteins, under basal conditions, or after IR (6 h) were analyzed by western blot. Images are representative of three independent experiments. Actin was used as a loading control. **b**, **c** Six hours and 24 h post-irradiation, γH2AX staining was performed and quantified using flow cytometry. Quantifications of three experiments are presented as means ± SD. **0.001 < *p* < 0.01; *0.01 < *p*< 0.05

IR is known to induce DNA damages leading to lethal cytotoxicity and a high activation of the DNA damage repair (DDR) is an important component of radioresistance of GBM and GSC in particular^18–20^. Therefore, we hypothesized that downregulation of α6-integrin gene expression might increase IR-induced DNA damage. To analyze this possibility, we determined whether inhibition of α6-integrin gene expression affected IR-induced phosphorylation of H2AX (γ-H2AX), a marker of DNA breaks. As expected, γ-H2AX staining increased rapidly following IR and returned to basal level at 24 h in control cells, indicating an efficient DDR process in GBM neurospheres (Fig. 4a, b). However, in cells transfected with an α6-integrin siRNA the DNA damages persisted significantly 24 h post-IR (Fig. 4a, b). In addition, we also observed at 6 h post-IR a higher increase of γ-H2AX staining in cells where α6-integrin expression was blocked compared with those transfected with the control siRNA.

CHK1 and CHK2 are two critical effector kinases involved in DNA damage checkpoint control and repair^20^. In particular, CHK1, through the phosphorylation of Cdc25c is known to control the G2/M checkpoint in response to DNA damage by IR. In addition, knockdown of CHK1 has been shown to increase cell death in response to double-strand breaks^21,22^. We therefore examined the status of these different proteins in GBM neurospheres when α6-integrin was downregulated. In cells expressing an α6-integrin siRNA, the expression and the phosphorylation of both CHK1 and Cdc25c were downregulated. In contrast, the α6-integrin siRNA did not affect the expression of CHK2 or its phosphorylation induced by IR (Fig. 4c). Upstream of CHK1 and CHK2, we also analyzed the two kinases ATR and ATM. We observed an increase in ATM phosphorylation in response to IR, whereas ATR seems to be constitutively phosphorylated. However, the phosphorylation and the expression of both kinases were not affected when α6-integrin expression was blocked (Fig. 4c).

### ZEB1 is involved in radioresistance mediated by α6-integrin overexpression in GBM

**Fig. 5:**
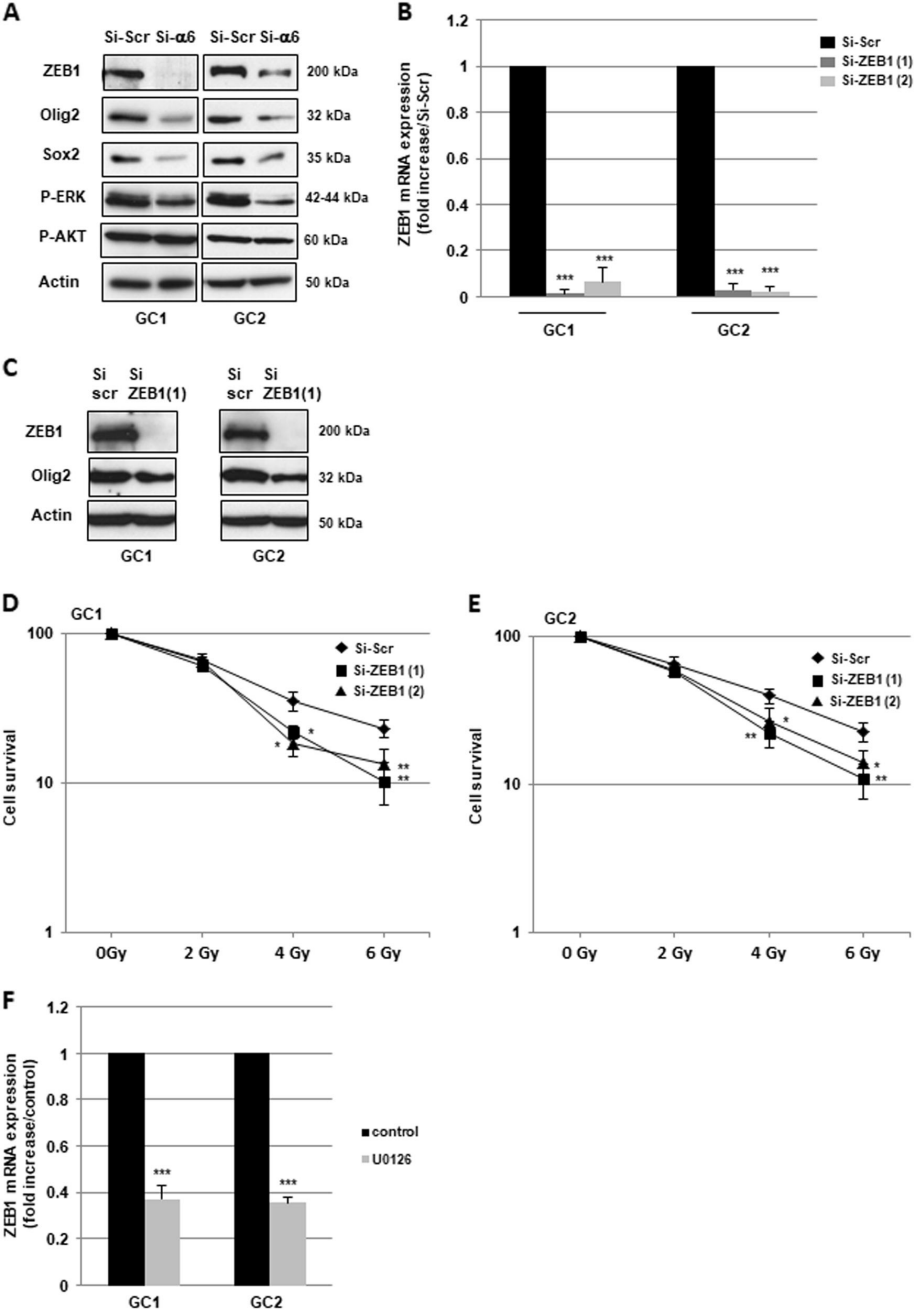
ZEB1 is involved in radioresistance mediated by α6-integrin overexpression in GBM. **a**-**e** Cells derived from two GBM biopsy specimens (GC1 and GC2) were transfected with an α6-integrin siRNA (si-α6), a ZEB1 siRNA (si-ZEB1 (1) and si-ZEB1 (2)), or a scramble control (si-Scr). **a**, **c** Different proteins, under basal conditions, were analyzed by western blot. Images are representative of three independent experiments. Actin was used as a loading control. **d**, **e** Cells were analyzed in 3D clonogenic assays as described in Materials and methods section. **f** GC1 and GC2 cells were pre-treated or not with 10 µM of the MEK inhibitor U-0126 and ZEB1 mRNA expression was analyzed by real-time PCR. Quantifications of three experiments are presented as means ± SD. ***p < 0.001; **0.001 < *p* < 0.01; *0.01 < *p* < 0.05

The epithelial–mesenchymal transition (EMT), originally observed during embryogenesis, also plays an important role in tumor progression, metastasis, cancer stem cell properties, drug resistance and radioresistance in multiple cancers^23,24^. The main transcription factors, which regulate EMT include ZEB1, ZEB2, snail, twist and slug. Therefore, to determine whether α6-integrin contributes to GBM radioresistance through the regulation of one of these transcription factors, we analyzed their expression in GBM neurospheres transfected with an α6-integrin siRNA or a scramble control. Only the expression of ZEB1 mRNA was significantly decreased (Supplemental Fig. S3B,C) in cells transfected with the α6-integrin siRNA compared with the control cells. This result was confirmed at the protein level. Indeed, a high inhibition of ZEB1 expression was observed by western blot when α6-integrin gene expression was downregulated (Fig. 5a). In addition, the knockdown of ZEB1 expression using two different specific siRNA (Si-ZEB1 (1) and Si-ZEB1 (2), Fig. 5b, c) mimics α6-integrin downregulation, by increasing radiosensitivity of neurospheres derived from human GBM biopsy specimens (Fig. 5d, e).

Integrins signaling in cancer cells has been extensively studied and two major pathways, the ERKs (Extracellular signal-regulated kinases) pathway and the PI3-kinase Pphosphatidylinositol 3 kinase)/AKT pathway, have been shown to mediate gene expression induced by integrins. As shown in Fig. 5a, both pathways are highly activated in GBM neurospheres transfected with the scramble control. However, only the ERKs phosphorylation was inhibited in cells transfected with the α6-integrin. We therefore analyzed the involvement of the ERKs in ZEB1 expression by using U-0126, an inhibitor of this pathway. In cells pre-treated with 10 µM of the inhibitor, a dose, which drastically inhibits the ERKs phosphorylation (Supplemental Fig. S4), we observed a significant decrease in ZEB1 expression (Fig. 5f).

It was recently reported that ZEB1 belongs to a network of three transcriptional factors including OLIG2 and SOX2 that represents a crucial driver of GBM^25^. Thus, we tested whether α6-integrin, in addition to ZEB1 could also regulate the expression of the two other members of this transcriptional network, OLIG2 and SOX2. As shown in Fig. 5a, we found a strong inhibition in OLIG2 and SOX2 protein expression in neurospheres transfected with the specific α6-integrin siRNA compared with the control siRNA. We also observed that ZEB1 downregulation by specific siRNA resulted in a significant decrease of OLIG2 expression (Fig. 5c), whereas SOX2 expression was not affected (data not shown).

### High expression of the five genes signature: α6-integrin/ZEB1/SOX2/OLIG2/CHK1 is prognostic of overall survival of GBM patients

**Fig. 6:**
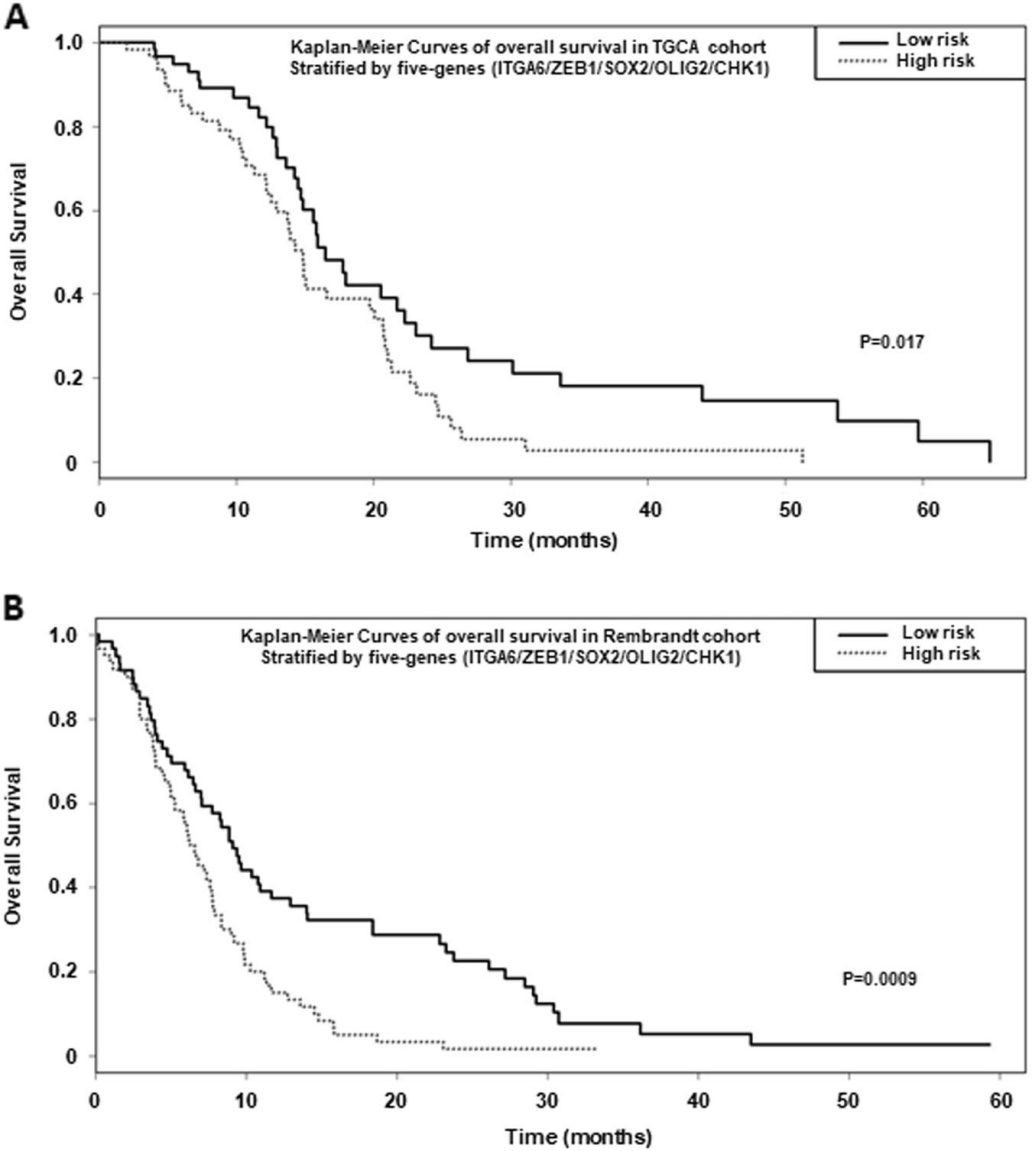
High expression of the five genes signature: α6-integrin/ZEB1/SOX2/OLIG2/CHK1 is prognostic of survival of GBM patients. Kaplan–Meier curves depict the probability of overall survival based on the expression of a five genes signature: α6-integrin/ZEB1/SOX2/OLIG2/CHK1. Analysis was performed from TGCA **a** or Rembrandt **b** databases. Patients were stratified by the upper or lower terciles and the statistical analysis performed as described in Materials and methods section

Finally we queried if the five genes signature: α6-integrin/ZEB1/SOX2/OLIG2/CHK1 correlates with survival of GBM patients. For this purpose, we analyzed two different databases, TCGA and Rembrandt. In the TGCA database, we focused on patients treated with standard radio-chemotherapy for primary GBM, excluding patients with prior glioma history (*n* = 184). Based on a Cox model, risk score and risk groups for the five genes signature were significantly associated with overall survival (risk score: hazard ratio (HR) = 2.72 [1.34; 5.51], *p* = 0.005; high versus low risk: HR = 1.74 [1.10; 2.73], *p* = 0.017) when patients samples were stratified by the highest and lowest terciles (Fig. 6a and b).

**Fig. 7:**
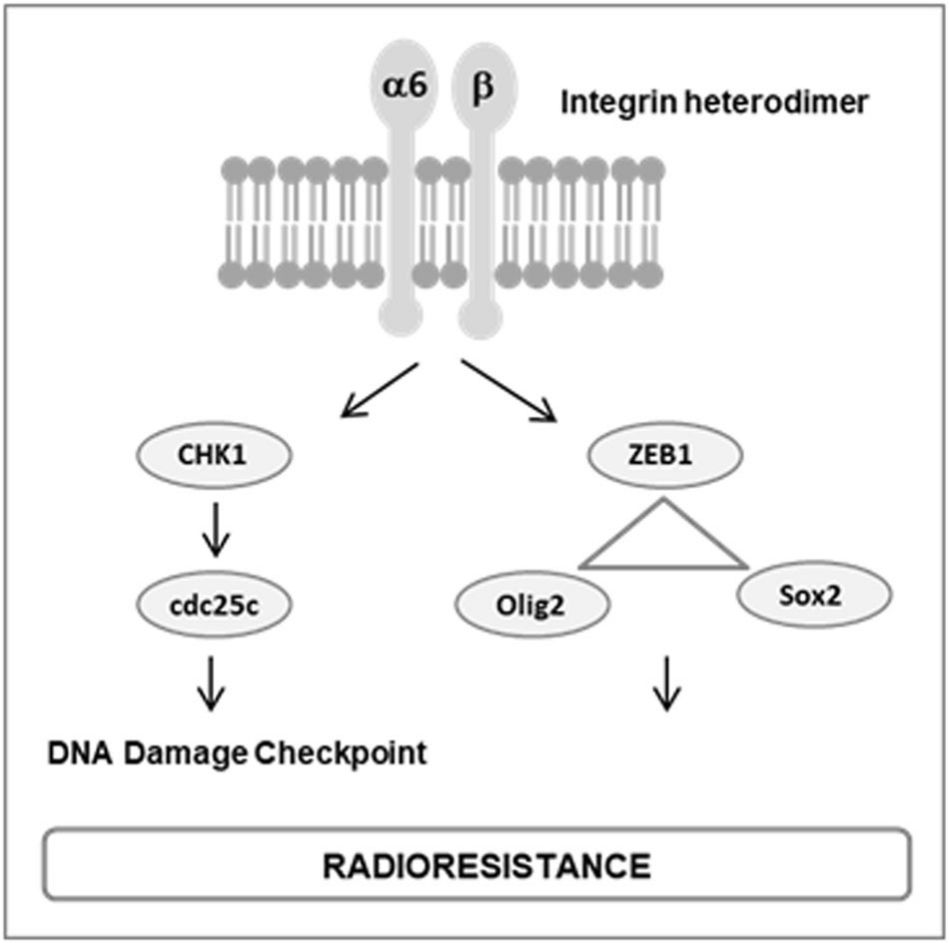
Proposed mechanism for the radio-protective effect of α6-integrin in GBM. We showed that α6-integrin contributes to GBM radioresistance by: controlling DNA damage checkpoint and repair via CHK1 and Cdc25c and regulating the transcriptional network ZEB1/OLIG2/SOX2

In the Rembrandt database (*N* = 178), the risk score and risk groups based on the same five genes were also statistically significant (risk score: HR = 2.72 [1.56; 4.75], *p* = 0.0004; high versus low risk: HR = 1.92 [1.31; 2.82], *p* = 0.0009, Fig. 6b), indicating that higher levels of this five genes signature predicted significantly shorter overall survival as compared with patients with lower expression.

## Discussion

Radiotherapy is the cornerstone of GBM standard treatment. However, the intrinsic radioresistance of cancer cells or the resistance acquired during the treatment by adaptation mechanisms leads to an inevitable recurrence of the disease with a median survival time of approximately 20 months^1^. Therefore, understanding the radioresistance mechanisms could provide novel potential therapeutic targets for GBM. In this study, we demonstrated for the first time that α6-integrin may be a novel key molecule responsible for radioresistance of GBM patients. We showed that targeting α6-integrin in cells derived from GBM biopsy specimens cultured as neurospheres, which express high levels of this particular integrin, sensitized cells to radiations. In cells downregulated for α6-integrin gene expression, we observed a decrease in cell survival after IR and an increase in radio-induced cell death. These results are in accordance with a previous report, which showed that the presence of laminin α2, a ligand of integrins (including α6-integrin) attenuates radiation-induced GBM stem cell death^26^. Radiation therapy is known to cause cell death by inducing DNA breaks. The importance of DNA repair and DNA damage checkpoint regulation as radioresistance mechanisms are recognized in GBM cells^20^. Here, we demonstrated that blocking α6-integrin expression in GBM cells can affect both, DNA damage checkpoint and repair. Indeed in the context of α6-integrin inhibition, we observed a persistence of γ-H2AX staining after IR, indicating that α6-integrin depleted cells were less able to repair DNA breaks. In addition, whereas IR induced the G2/M cell cycle checkpoint in control cells, blocking α6-integrin expression resulted in the abrogation of DNA damage-induced cell cycle arrest. The checkpoint kinase CHK1 and its downstream target Cdc25c are known to regulate, in particular, the G2/M arrest. In addition, knockdown or inhibition of CHK1 increases cell death in response to double-strand breaks. In accordance with this mechanism, we demonstrated that targeting α6-integrin in GBM cells, drastically decreased the expression of both CHK1 and Cdc25c.

Therefore, α6-integrin overexpression in GBM cells may contribute to the efficiency of DNA damage response leading to radioresistance (figure 7). These results are in accordance with previously reported data, which showed that combined inhibition of DNA repair and cell cycle checkpoint targets, such as CHK1, are particularly effective to overcome radioresistance of GCS^18^.

Recently, Hu et al. have also shown that α6-integrin blocking radiosensitizes breast cancer cells. In contrast to what we observed in GBM, they reported a mechanism that involves an increase in cell apoptosis^27^.

Specific EMT inducers have been associated with cancer stem cell properties and anticancer therapies resistance^23,24,28–30^. In particular, it is interesting to note that the transcription factor ZEB1, rather than EMT itself, has been involved in radioresistance of prostate and breast tumors^31,32^. In GBM cells, we showed in the present study that blocking α6-integrin downregulated ZEB1 protein expression without affecting the other major EMT regulators. Integrins signaling is known to regulate gene expression in particular via the activation of AKT and ERKs^33^. In addition, a number of studies have reported the regulation of ZEB1 expression in tumor cells through different signaling cascades including the AKT and ERKs pathways^34^. Here we showed that α6-integrin could regulate ZEB1 expression in GBM by an ERK-dependent mechanism.

A recent study provided evidence that ZEB1 could control invasion, tumorigenesis and chemoresistance in GBM^30^. In the present study, we demonstrated for the first time an important role of this transcription factor in radioresistance of GSC-enriched neurospheres derived from human GBM. Therefore, α6-integrin may contribute to radioresistance of GBM partly through the regulation of ZEB1 expression.

Recently, the transcriptional network ZEB1/OLIG2/SOX2 has been shown to be crucial in GBM irrespective of driver mutations, playing an important role in tumor initiation, stemness properties, proliferation and tumorigenicity^25^. Interestingly, OLIG2 and SOX2 have also been previously involved in radioresistance of GBM^35–37^. Here we provide evidences that α6-integrin may be a target with a great therapeutic interest in GBM as we demonstrated that this integrin, overexpressed in GBM, control the expression of the transcriptional network ZEB1/OLIG2/SOX2 (figure 7).

Finally, consistent with our pre-clinical data in vitro, the analyses from two distinct clinical datasets, TGCA and Rembrandt databases, demonstrate that GBM patients with high levels of the five genes signature, including α6-integrin and its targets, CHK1, ZEB1, OLIG2 and SOX2, have a significantly shorter overall survival as compared with patients with lower expression.

Our study not only reveals a novel mechanism of GBM radioresistance in which α6-integrin plays a critical role but also open a new potential therapeutic strategy involving α6-integrin targeting in association with radiation therapy to overcome GBM radioresistance.

## Materials and methods

### GBM patient-derived cells

All GBM specimens were obtained after written informed consent from patients admitted to the Neurosurgery Department at Toulouse University Hospital under a clinical protocol approved by the Human Research Ethics Committee. Patient brain tumor samples were classified as GBM based on the WHO. The GBM samples were processed as described by Avril et al.^38^ to obtain primary neurospheres cell lines. Neurospheres were maintained in Dulbecco’s modified Eagle’s medium-F12 (GIBCO) supplemented with B27 and N2 (Life Technologies), 25 ng/ml of Fibroblast Growth factor-2 (FGF-2) and Epidermal growth factor (EGF) (Peprotech) at 37 °C in a 5% CO_2_ humidified incubator and cultured during <12 passages to avoid loss of cell characteristics.

### Cells IR

Cells cultured as specifically described for each method were exposed to different doses of radiation (2–10 Gy) as indicated, using an Irradiator Gamma-cell Exactor 40 (Nordion, Ottawa, ON, Canada).

### SiRNA transfection, RNA extraction, reverse transcription and real-time PCR

The siRNA directed against α6-integrin, ZEB1 or the scramble control were purchased from Qiagen and transfected using Lipofectamine RNAi Max (Invitrogen) following the manufacturer's protocol. Total RNA was isolated by the RNeasy RNA isolation Kit (Qiagen) then reverse transcribed using the RT transcription kit Prime Script RT Reagent kit (TAKARA). mRNA expression was determined by real-time PCR, using the ABI-Stepone + (Applied Biosystems). GAPDH was used for normalization.

### 3D clonogenic assay

Cells derived from GBM biopsy specimens, transfected with a specific α6-integrin siRNA, a specific ZEB1 siRNA or a scramble control were seeded in 96 wells plates (100 cells per wells, 12 wells per condition) for 24 h then treated or not with different doses of gamma rays (2–6 Gy). After 8–10 days, the number of neurospheres per well with >50 cells were counted under the microscope. The surviving fraction was calculated taking into account the plating efficiency in the non-irradiated condition (PE = spheres number/seeded cells number × 100).

### Western blot analysis

Identical levels of proteins were separated by sodium dodecyl sulfate–polyacrylamide gel electrophoresis and analyzed by western blot with the indicated antibodies as described previously^39^. Antibodies used for western blot: SOX2, Tuj-1 (Abcam), OLIG2, Nestin, actin, GFAP (Millipore), ZEB1, ZEB2, CHK1, CHK2, Cdc25c, phospho-Cdc25c and phosphor-ERK (Cell Signaling).

### **α**6-Integrin protein expression by FACS analysis

α6-Integrin protein expression on cells derived from GBM biopsy specimens was analyzed using the Flow cytometry live cell protocol from Cell Signaling Technology and the PE-conjugated anti-α6-integrin antibody from eBioscience. Stained cells were analyzed by flow cytometry (BD Accuri^TM^ C6 cytometer) and the data were analyzed by the BD Accuri C6 software. The specific florescent index (SFI) was calculated as previously described, with the following formula:

SFI = (MFI antibody − MFI isotype control)/MFI isotype control (MFI: mean fluorescent index).

### γH2AX staining

Cells derived from GBM biopsy specimens, transfected with a specific α6-integrin siRNA or a scramble control were exposed to a radiation dose of 4 Gy. Six hours or 24 h post-IR, cells were fixed and intracellular staining with an Alexa Fluor 488 anti-H2AX-Phosphorylated (Ser139) antibody performed according to the manufacturer's protocol (BioLegend). Stained cells were analyzed by flow cytometry (BD Accuri^TM^ C6 cytometer) and the data were analyzed by the BD Accuri C6 software.

### Apoptosis

Annexin V/propidium iodide (PI) staining was performed using an apoptosis kit from Affimetrix, eBioscience, according to the manufacturer's protocol. Cells were analyzed 24 h to 48 h post-IR by flow cytometry (BD Accuri^TM^ C6 cytometer) and the flow cytometry data were analyzed by the BD Accuri C6 software.

### Cell cycle analysis

Forty-eight hours post-IR, cells were fixed in 70% ice-cold ethanol for 1 h at 4  °C. After washing, the cell pellet was resuspended in PI-staining buffer (50 μg/ml PI, 10 μg/ml RNAse A) and incubated for 15 min at 37  °C. The DNA content was analyzed by flow cytometry (BD Accuri^TM^ C6 cytometer). Percentage of cells in sub-G1, G1/S and G2/M phases were quantified by the BD Accuri C6 software.

### Correlation of five genes signature: α6-integrin/ZEB1/SOX2/OLIG2/CHK1 with survival of GBM patients

For survival analysis, using the GBM database of the Cancer Genome Atlas, (TCGA) (https://genome-cancer.ucsc.edu/), we focused on patients treated with standard radio-chemotherapy for primary GBM, excluding patients with prior glioma history (*n* = 184). Overall survival rates were estimated using Kaplan–Meier method and univariate analyses were performed using Cox proportional hazard model. From predictors included in a Cox model, a risk score prediction was created. It is based on the linear predictor given by the model. This score was then divided into three groups by taking the terciles of the risk score. Thus, three groups were established (poor, medium and good prognostic) and correspond to the signature for this dataset. To confirm the prognostic ability of our predictors, the same strategy was applied on REMBRANDT dataset (*n* = 178) (http://www.betastasis.com/glioma/rembrandt/). All reported *p*-values were two-sided. For all statistical tests, differences were considered significant at the 5% level. Statistical analysis was performed using R. 3.4.2 software.

## Electronic supplementary material

supplemental figure S1

supplemental figure S2

supplemental figure S3

supplemental figure S4

Supplementary figure legends

## References

[1] Weller M (2017). Rindopepimut with temozolodide for patients with newly diagnosed, EGFRVIII-expressing glioblastoma (ACT IV): a randomised, double-blind, international phase 3 trial. Lancet Oncol..

[2] Wen PY, Kesari S (2008). Malignant gliomas in adults. N. Engl. J. Med..

[3] Meldolesi J (2016). Pharmacology of the cell/matrix form of adhesion. Pharmacol. Res..

[4] Bianconi, D., Unseld, M. & Prager, G. W. Integrins in the Spotlight of Cancer. *Int. J. Mol. Sci*. **17**, 2037 (2016). 10.3390/ijms17122037 PMC5187837 27929432

[5] Gingras MC, Roussel E, Bruner JM, Branch CD, Moser RP (1995). Comparison of cell adhesion molecule expression between glioblastoma multiforme and autologous normal brain tissue. J. Neuroimmunol..

[6] Previtali S (1996). Alpha6 beta 4 and alpha 6 beta 1 integrins in astrocytomas and other CNS tumors. J. Neuropathol. Exp. Neurol..

[7] Hall PE, Lathia JD, Miller NG, Caldwell MA, French-Constant C (2006). Integrins are markers of human neural stem cells. Stem Cells.

[8] Krebsbach PH, Villa-Diaz LG (2017). The role of Integrinalpha6 (CD49f) in stem cells: more than a conserved biomarker. Stem. Cells Dev..

[9] Lathia JD (2010). Integrin alpha 6 regulates glioblastoma stem cells. Cell. Stem. Cell..

[10] Velpula KK (2012). Glioma stem cell invasion through regulation of the interconnected ERK, integrin alpha 6 and N-cadherin signaling pathway. Cell. Signal..

[11] Brooks DL (2016). ITGA6 is directly regulated by hypoxia-inducible factors and enriches for cancer stem cell activity and invasion in metastatic breast cancer models. Mol. Cancer.

[12] Cariati M (2008). Alpha-6 integrin is necessary for the tumourigenicity of a stem cell-like subpopulation within the MCF7 breast cancer cell line. Int. J. Cancer.

[13] Ortiz-Sanchez E (2016). Characterization of cervical cancer stem cell-like cells: phenotyping, stemness, and human papilloma virus co-receptor expression. Oncotarget.

[14] Landowski TH (2014). Targeting integrin alpha 6 stimulates curative-type bone metastasis lesions in a xenograft model. Mol. Cancer Ther..

[15] Laudato S (2017). P53-induced miR-30e-5p inhibits colorectal cancer invasion and metastasis by targeting ITGA6 and ITGB1. Int. J. Cancer.

[16] Shaw LM, Chao C, Wewer UM, Mercurio AM (1996). Function of the integrin alpha 6 beta 1 in metastatic breast carcinoma cells assessed by expression of a dominant-negative receptor. Cancer Res..

[17] Maier, P., Hartmann, L., Wenz, F. & Herskind, C. Cellular pathways in response to ionizing radiation and their targetability for tumor radiosensitization. *Int. J. Mol. Sci*. **17**, 102 (2016). 10.3390/ijms17010102 PMC4730344 26784176

[18] Ahmed SU (2015). Selective inhibition of parallel DNA damage response pathways optimizes radiosensitization of glioblastoma stem-like cells. Cancer Res..

[19] Bao S (2006). Glioma stem cells promote radioresistance by preferential activation of the DNA damage response. Nature.

[20] Frosina G (2009). DNA repair and resistance of gliomas to chemotherapy and radiotherapy. Mol. Cancer Res..

[21] Bartek J, Lukas J (2003). Chk1 and Chk2 kinases in checkpoint control and cancer. Cancer Cell..

[22] Patil M, Pabla N, Dong Z (2013). Checkpoint kinase 1 in DNA damage response and cell cycle regulation. Cell. Mol. Life Sci..

[23] Garg M (2017). Epithelial plasticity and cancer stem cells: major mechanisms of cancer pathogenesis and therapy resistance. World J. Stem Cells.

[24] Kahlert UD, Joseph JV, Kruyt FA (2017). EMT- and MET-related processes in nonepithelial tumors: importance for disease progression, prognosis, and therapeutic opportunities. Mol. Oncol..

[25] Singh DK (2017). Oncogenes activate an autonomous transcriptional regulatory circuit that drives glioblastoma. Cell Rep..

[26] Lathia JD (2012). Laminin alpha 2 enables glioblastoma stem cell growth. Ann. Neurol..

[27] Hu T, Zhou R, Zhao Y, Wu G (2016). Integrin alpha 6/Akt/Erk signaling is essential for human breast cancer resistance to radiotherapy. Sci. Rep..

[28] Nantajit D, Lin D, Li JJ (2015). The network of epithelial-mesenchymal transition: potential new targets for tumor resistance. J. Cancer Res. Clin. Oncol..

[29] Zhang P, Sun Y, Ma L (2015). ZEB1: at the crossroads of epithelial-mesenchymal transition, metastasis and therapy resistance. Cell Cycle.

[30] Siebzehnrubl FA (2013). The ZEB1 pathway links glioblastoma initiation, invasion and chemoresistance. EMBO Mol. Med..

[31] El Bezawy R (2017). miR-875-5p counteracts epithelial-to-mesenchymal transition and enhances radiation response in prostate cancer through repression of the EGFR-ZEB1 axis. Cancer Lett..

[32] Zhang P (2014). ATM-mediated stabilization of ZEB1 promotes DNA damage response and radioresistance through CHK1. Nat. Cell Biol..

[33] Paolillo M, Serra M, Schinelli S (2016). Integrins in glioblastoma: still an attractive target?. Pharmacol. Res..

[34] Tania M, Khan MA, Fu J (2014). Epithelial to mesenchymal transition inducing transcription factors and metastatic cancer. Tumour Biol.: J. Int. Soc. Oncodev. Biol. Med..

[35] Garros-Regulez L (2016). Targeting SOX2 as a therapeutic strategy in glioblastoma. Front. Oncol..

[36] Lee Y (2015). FoxM1 promotes stemness and radio-resistance of glioblastoma by regulating the master stem cell regulator Sox2. PLoS One.

[37] Mehta S (2011). The central nervous system-restricted transcription factor Olig2 opposes p53 responses to genotoxic damage in neural progenitors and malignant glioma. Cancer Cell..

[38] Avril T (2012). Human glioblastoma stem-like cells are more sensitive to allogeneic NK and T cell-mediated killing compared with serum-cultured glioblastoma cells. Brain. Pathol..

[39] Kowalski-Chauvel A (2017). Targeting progastrin enhances radiosensitization of colorectal cancer cells. Oncotarget.

