# Supplementary figures and images for: Alpha-6 integrin promotes radioresistance of glioblastoma by modulating DNA damage response and the transcription factor Zeb1
Source: Cell Death Dis. 2018 Aug 29;9(9):872. doi: 10.1038/s41419-018-0853-x (PMC6115442; doi:10.1038/s41419-018-0853-x)

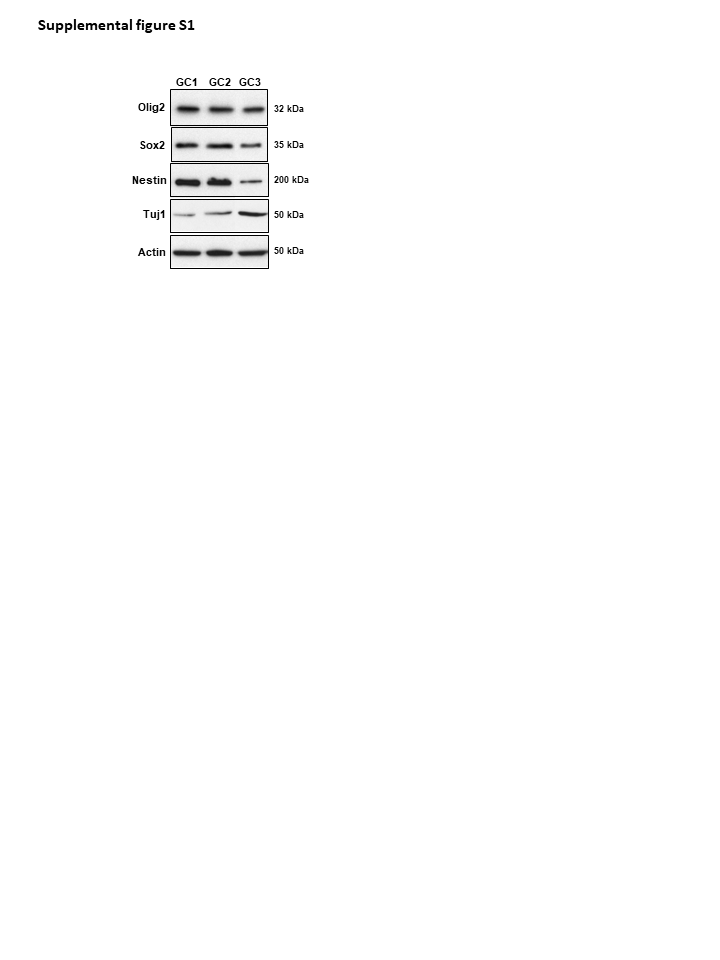

Supplement: Supplementary file 1 — supplemental figure S1 [file 41419_2018_853_MOESM1_ESM.tif]

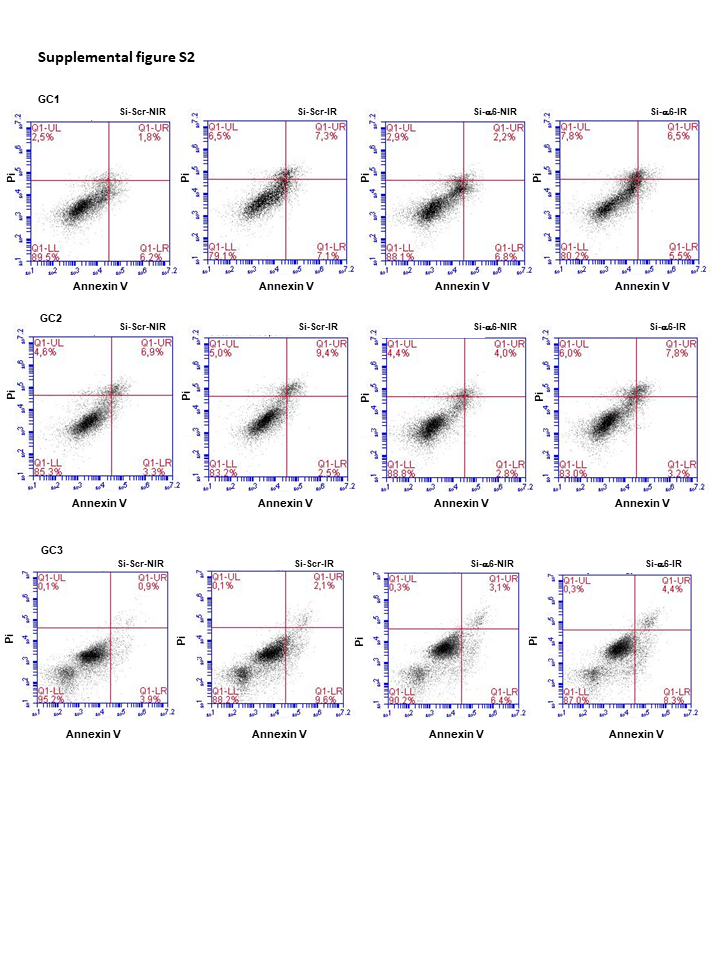

Supplement: Supplementary file 2 — supplemental figure S2 [file 41419_2018_853_MOESM2_ESM.tif]

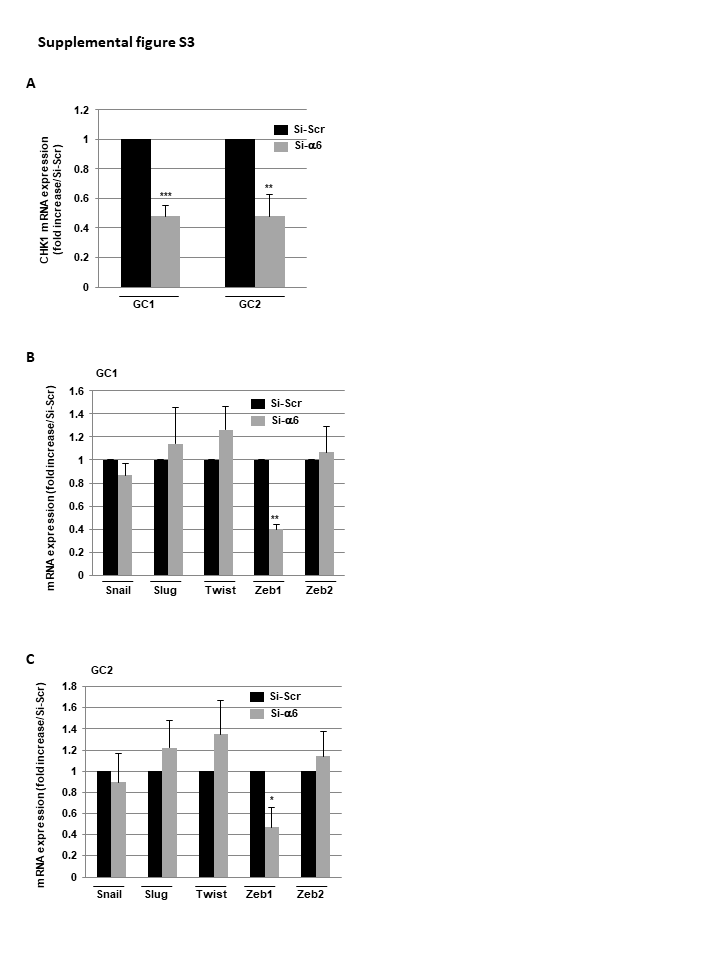

Supplement: Supplementary file 3 — supplemental figure S3 [file 41419_2018_853_MOESM3_ESM.tif]

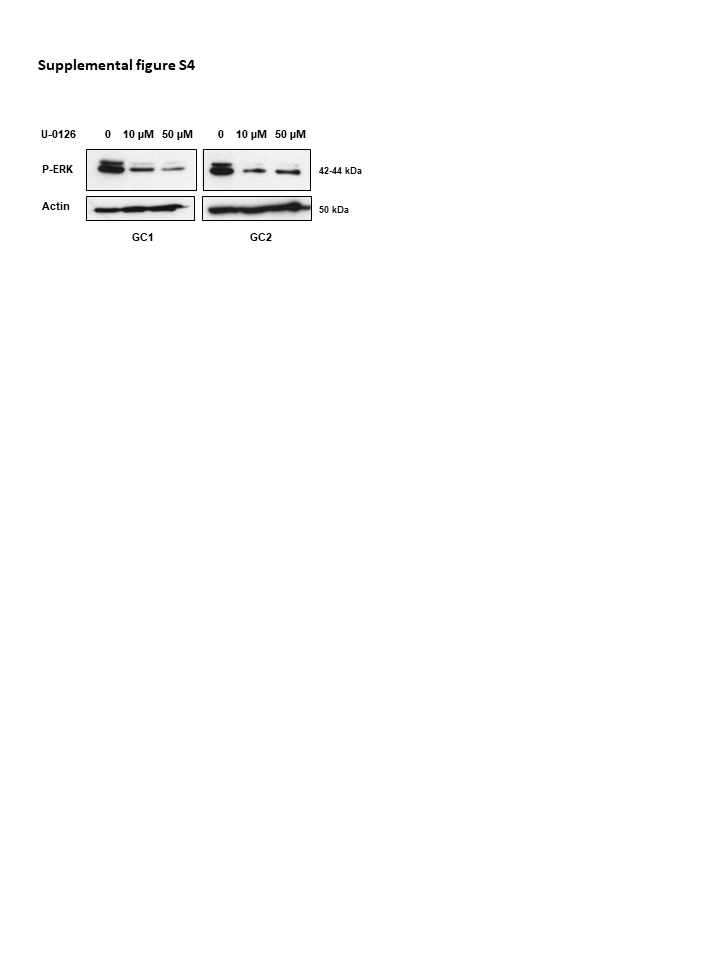

Supplement: Supplementary file 4 — supplemental figure S4 [file 41419_2018_853_MOESM4_ESM.tif]
