# Supplementary material for: Alpha-6 integrin promotes radioresistance of glioblastoma by modulating DNA damage response and the transcription factor Zeb1
Source: Cell Death Dis. 2018 Aug 29;9(9):872. doi: 10.1038/s41419-018-0853-x (PMC6115442; doi:10.1038/s41419-018-0853-x)
Supplement: Supplementary file 5 — Supplementary figure legends [file 41419_2018_853_MOESM5_ESM.docx]

**SUPPLEMENTAL FIGURES LEGENDS**

**Supplemental Fig. 1** **Expression of Stem cell and differentiation markers in Tumor cells derived from human glioblastoma biopsy specimens.** The expression of stem cell (Olig2, Sox2, Nestin) and differentiation (Tuj1) markers was analysed by western blot in tumor cells derived from 3 human GBM biopsy specimens (GC1, GC2 and GC3) cultured as neurospheres. Images are representative of 3 independent experiments. Actin was used as a loading control.

**Supplemental Fig. 2 Targeting α6-integrin expression do not increase apoptosis.** Cells derived from 3 GBM biopsy specimens (GC1, GC2, GC3) were transfected with an α6-integrin siRNA (si-α6) or a scramble control (si-Scr). Annexin V/Pi staining was analysed by flow cytometry in cells non-irradiated (NIR) or 48h post-irradiation (6 Gy, IR). Images are representative of 3 independent experiments.

**Supplemental Fig. 3 Down-regulation of α6-integrin decreases CHK1 and ZEB1 mRNA expression.** Cells derived from 2 GBM biopsy specimens (GC1 and GC2) were transfected with an α6-integrin siRNA (si-α6) or a scramble control (si-Scr). Expression of different mRNA was analyzed by real-time PCR. Quantifications of 3 experiments are presented as means ± SD. ***P<0.001; **0.001<P<0.01; *0.01<P<0.05.

**Supplemental Fig. 4 Inhibition of the ERKs phosporylation by the MEK inhibitor.** GC1 and GC2 cells were pretreated or not for 24h with 10 or 50 µM of the MEK inhibitor U-0126. ERKs phosporylation was analysed by western blot using an anti-phospho-ERK antibody. Actin was used as a loading control. Images are representative of 3 independent experiments
